# QM/MM Molecular Dynamics Study of the Galactopyranose → Galactofuranose Reaction Catalysed by *Trypanosoma cruzi* UDP-Galactopyranose Mutase

**DOI:** 10.1371/journal.pone.0109559

**Published:** 2014-10-09

**Authors:** Gustavo Pierdominici-Sottile, Rodrigo Cossio Pérez, Johan F. Galindo, Juliana Palma

**Affiliations:** 1 Departamento de Ciencia y Tecnología, Universidad Nacional de Quilmes, Bernal, Argentina; 2 Quantum and Computational Chemistry Group, Departamento de Química, Universidad Nacional de Colombia, Bogotá, Colombia; University of Cantebury, New Zealand

## Abstract

The enzyme UDP-Galactopyranose Mutase (UGM) catalyses the conversion of galactopyranose into galactofuranose. It is known to be critical for the survival and proliferation of several pathogenic agents, both prokaryotic and eukaryotic. Among them is *Trypanosoma cruzi*, the parasite responsible for Chagas' disease. Since the enzyme is not present in mammals, it appears as a promising target for the design of drugs to treat this illness. A precise knowledge of the mechanism of the catalysed reaction would be crucial to assist in such design. In this article we present a detailed study of all the putative steps of the mechanism. The study is based on QM/MM free energy calculations along properly selected reaction coordinates, and on the analysis of the main structural changes and interactions taking place at every step. The results are discussed in connection with the experimental evidence and previous theoretical studies.

## Introduction

Chagas' disease, also known as American trypanosomiasis, affects approximately 8 million people worldwide. It is endemic in Latin America but in the last decades it has also spread towards North America and Europe [1]. Its pathogenic agent is the flagellate protozoan *Trypanosoma cruzi* (*T. cruzi*), which is transmitted to humans by the faeces of triatomine insects. The disease was first described by Dr. Carlos Chagas in Brazil in 1909. Despite this early discovery there are still no drugs capable of curing it. Nifurtimox and Benznidazole are used in the acute phase of the disease. However none of them are efficient and both have strong side effects [2]–[4]. Most patients discontinue the treatment when the side effects become too severe. For these reasons new and more efficient drugs are needed.

Galactose is a common monosaccharide. In mammals it is exclusively found as galactopyranose (Gal*p*), the six-membered ring hemiacetal form. On the other hand, in *T. cruzi* and many other human pathogens such as *Mycobacterium tuberculosis*, *Escherichia coli*, *Leishmania major*, *Aspergillus fumigatus*, *Salmonella typhimurium* and *Klebsiella pneumoniae* [5]–[8], it is found as galactofuranose (Gal*f*), the five-membered ring hemiacetal form [5], [ 6], [ 8]–[17]. The sole source of Gal*f* in these species is the enzyme UDP-Galactopryranose Mutase (UGM), which catalyses the isomerization between UDP-Gal*p* and UDP-Gal*f*, the precursor of Gal*f* [18], [ 19]. It is known that Gal*f* is an essential component of the cell wall and the extracellular matrix of these pathogens [13], [ 19]. Suppression of the UGM gene in many of them caused attenuated virulence and increased sensitivity to drugs [20]–[22]. In *T. cruzi*, particularly, Gal*f* is attached to glycoinositolphospholipids and glycosylphosphatidylinositol anchor proteins [23], [ 24], which are highly expressed throughout the life cycle of the parasite and are essential for its survival and proliferation [25]–[27]. When *T. cruzi* is incubated with specific antibodies against Gal*f*, the binding of the parasite to the mammalian cells is blocked, leading to an 80% decrease in infectivity [13]. Since neither Gal*f* nor UGM have ever been found in mammals, UGM has gathered significant interest as a target for drugs design [28]. Due to this interest, it has been subjected to several structural and mechanistic studies [28]–[30].

In 2001 was presented the first known crystallographic structure of a UGM. It corresponded to *E. coli*, [31]. After that, other bacterial structures were also determined [31]–[36]. Eukaryotic UGMs received less attention. The first structure of that kind, corresponding to *Aspargillus fumigatus*, was published in 2012 [37]. Shortly after, the one of *T. cruzi* (*Tc*UGM) became also available [38]. The comparison between eukaryotic and prokaryotic UGMs revealed that they share a common folding and a GxGxxG motif, necessary to bind the cofactor, flavin adenine dinucleotide (FAD) [39]. Moreover, the cofactor conformation and its interaction with the enzyme environment is highly conserved in both groups. However, the interactions with the substrate differ significantly and the sequence identity is pretty low (15%) [38]. In the active site, only 5 out of 13 residues are shared. Besides eukaryotic UGMs are approximately 100 residues longer than prokaryotic ones. This additional part of the chain forms extra secondary structures, modifying the active site flexibility and the oligomerization state of the enzyme [39].

**Figure 1:**
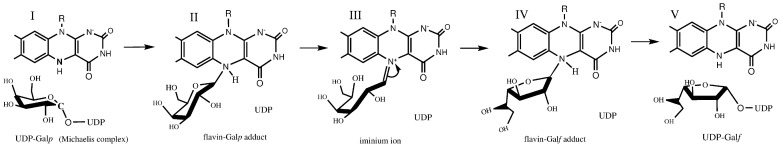
Main species of the reaction mechanism.

**Figure 2:**
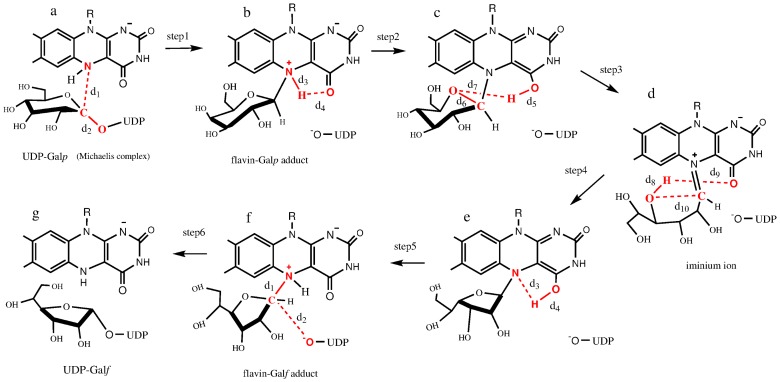
Detailed mechanism for the reaction catalysed by *Tc*UGM. The mechanism includes the intermediates detected by experiments as well as those whose existence was inferred from theoretical considerations. Red color is used to denote the bonds being broken (solid line) or formed (dashed line), as well as the atoms involved. The distances between these atoms are labelled because they are used to define the reaction coordinates.

Fig. 1 shows the main species of the catalysed reaction. The transformations between these species we will be denoted as “stages” of the mechanism. The first and last stages consist of just one reaction step while the second and third stages involve two. All the steps of the mechanism under analysis are presented in Fig. 2. According to different experimental studies the reaction initiates with the formation of a flavin-galactose adduct (conversion from I to II in Fig. 1) [28], [ 34], [ 40], [ 41]. This requires the rupture of the Gal*p*-UDP bond and the creation of a bond between Gal*p* and the nitrogen at position 5 of the reduced flavin adenine dinucleotide (FADH), N5_FADH_ [40], [ 42]–[44].

It was experimentally found that no conversion between Gal*p* and Gal*f* occurred when the native cofactor was replaced by 5-deaza-FAD [45]. Since this modified cofactor can only participate in two-electron transfers, it was argued that the mechanism in UGM should involved a one electron transfer. In particular, it was suggested that an oxocarbenium ion was first formed, followed by a single electron transfer, and that the recombination of the radicals so formed would produce the flavin-galactose adduct. However, it was then argued that the evidence presented does not exclude the possibility of a nucleophilic attack of N5_FADH_ onto the anomeric C of Gal*p*, C1X_GAL_, with a 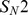 type mechanism [46]. Positional isotope effects experiments, together with studies that employed FAD analogues with different electron density on N5_FADH_, uphold this hypothesis [46]. Besides, the analysis of the crystallographic structures, as well as recent investigations on *Tc*UGM, give further support to this mechanism [28], [ 34], [ 40], [ 43]. The next stage (conversion from II to III in Fig. 1), involves the opening of the ring to form an iminium ion [40], [ 43]. This intermediate species has been trapped using NaCNBH_3_ in two independent studies [28], [ 40]. Naively, one would suggest that the iminium is formed by a direct proton transfer from N5_FADH_ to the cyclic oxygen of galactose, O5X_GAL_. However, as noted by Huang *et. al.*, such transference involves the passage through a four-membered ring structure which is rather high in energy. As an alternative, the same authors proposed that the proton is first passed from N5_FADH_ to O4_FADH_, and then transferred to Gal*p* to initiate the opening of the ring [41]. Once the iminium intermediate is formed, two stages are needed to complete the reaction. They can be considered as the reverse of the two previous stages, except for the fact that galactose is now in the furanose form. Thus, stage three involves the sugar ring closure to form Gal*f* (conversion from III to IV in Fig 1). Sobrado *et. al.* indicated that this is the stage that determines the rate of the whole process [28]. Stage four consists of the breaking of the flavin-substrate bond along with the binding of UDP to the sugar (conversion from IV to V in Fig. 1).

Huang *et. al.* performed a theoretical study on the mechanism of the reaction catalysed by UGM [41]. They carried out electronic structure computations on active site models built from the PDB structure of *Klebsiella pneumoniae* UGM (*Kp*UGM). The largest of their models contained 26 active site residues plus the substrate, the cofactor and several crystallographic water molecules. A quantum mechanics-molecular mechanics level theory (QM/MM) was employed to characterize the structures of reactants, products, intermediate species and transitions states appearing in the mechanism. More recently, the involvement of several active site residues on the catalytic activity of *Tc*UGM was evaluated through site directed mutageneis experiments [29].

In this article we present a QM/MM molecular dynamics study of the reaction catalysed by *Tc*UGM. We applied the umbrella sampling technique to obtain the free energy profiles along different reaction coordinates, conveniently defined to describe every step of the mechanism. QM/MM free energy computations have become a widely employed tool to gain information on the atomistic details of enzymatic reactions. One of their main assets is the ability to reveal both, energetic and dynamical contributions to catalysis. We also analysed the most significant conformational changes and interactions taking place at each step. This includes the monitoring of bond distances, dihedral angles, H-bonds, partial charges, bond orders as well as the Cremer-Pople angles that describe the conformations of the pyranose and furanose rings [47]. Finally, we implemented an energy decomposition method to evaluate the contribution of the active site residues to the lowering of the barriers at every step. The results of the simulations are discussed in connection with previous experimental findings, as well as with the theoretical analysis of Huang *et. al*.

## Results and Discussion

**Figure 3:**
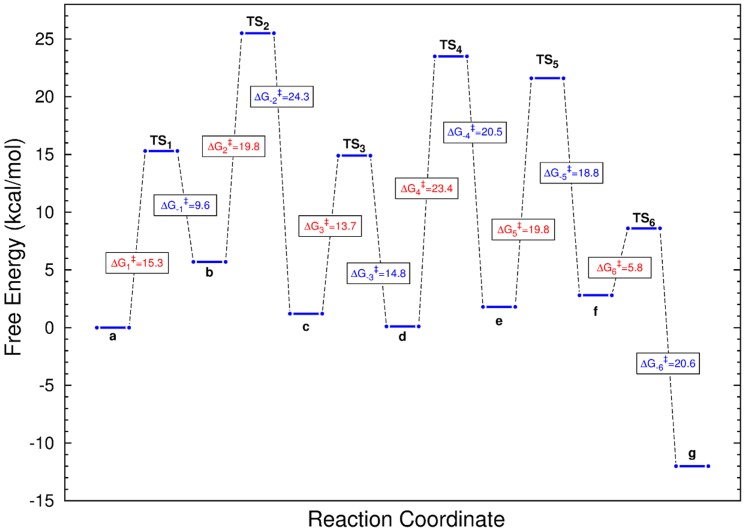
Sketch of the free energy profile for the whole mechanism. Activation free energies (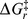) for all the steps of the mechanism under analysis. Data for the direct reaction are shown in red while those of the reverse reaction are shown in blue. Letters a to g denote the species presented in Fig. 2. The highest point of the profile corresponds to the transition state for the tautomerization of FADH (conversion from b to c of Fig. 2). The largest barrier corresponds to the ring closure into the furanose form (conversion from d to e of Fig. 2).

In Fig. 3 we present a sketch of the free energy changes (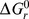) and free energy barriers (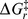) for the successive steps of the mechanism presented in Fig. 2. The profile shows that the barrier for the ring opening (step 3) is sensibly smaller than that of the ring closure (step 4). In fact, the barrier for step 4 is the highest. This is in agreement with the experimental findings of Sobrado *et. al.* [28]. The profile also indicates that products are more stable than reactants. The same result was found in the computations of Huang *et. al.* [41]. For the reverse reaction the largest barrier corresponds to the tautomerization of FADH. We also note that for both, forward and backward reactions, the appearance of the iminium ion species presents a small barrier.

In the following sections we describe in detail the outcome of the QM/MM computations for all the stages of the catalysed reaction. When pertinent, the results are compared with those recently reported for *Kp*UGM [41]. We note, however, that a meaningful comparison between these computations requires keeping in mind the aspects in which they differ. Among the differences we have: (1) that *Kp*UGM and *Tc*UGM bear low sequence homology: 18.3% for the whole protein and only 5 out of 13 residues for the active site [39]; (2) that free energy computations include dynamical effects that are not considered in electronic structure computations; (3) that residue His62 was protonated in the present work but was set as neutral in the work of Huang *et. al.*; (4) that we modelled the whole *Tc*UGM crystal structure in explicit solvent while Huang *et. al.* considered an active site model consisting of the cofactor, the substrate, 26 active site residues and 8 water molecules; (5) that the quantum subsystems and the DFT levels of theory employed in each computation were different.

In what follows the numbers of the residues and the names of the atoms correspond to the crystal structure of *Tc*UGM taken from the Protein Data Bank (PDB code 4DSH). Figure 2 should serve as a guide for the reading of the following sections. It describes every step of the mechanism, highlighting the distances involved in the definition of the corresponding reaction coordinates. On the other hand, at the supplementary information section, we provide pdb files with the average structures of reactants, products and all the intermediates of the reaction (Text S1 to Text S7). These structures can be used to obtain information that was not included in the main text in order to keep the article at a reasonable length. For the same reason, several pictures depicting the evolution of important distances and angles along the different steps of the reaction are given at the supplementary information section.

### Stage 1: Formation of the flavin-Gal*p* adduct

This stage consists of just one concerted step in which N5_FADH_ bonds covalently to the anomeric C of Gal*p* while the UDP moiety detaches from it. In panel a) of Figure S1 the evolution of the distances involved in the definition of the reaction coordinate (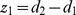) along this step, are shown. At the transition state, TS1, we obtained 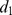  = 2.52 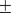 0.05 Å and 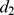  = 2.15 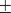 0.05 Å. This corresponds to bond orders of 0.30 and 0.19 for the C1X_GAL_-O3B_UDP_ and C1X_GAL_-N5_FADH_ bonds, respectively. These orders support the hypothesis that the reaction proceeds via a dissociative S*_N_*2 mechanism, as has been suggested by several independent experimental studies [28], [ 34], [ 46]. The calculated 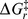 and 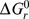 are 15.3 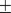 0.3 kcal/mol and 5.7 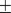 0.2 kcal/mol, respectively. These values qualitatively agree with those of Huang *et. al.* who found 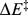  = 18.9 kcal/mol and 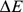  = 9.63 kcal/mol for the same process. The partial charges of key atoms for reactants (Michaelis complex), TS1 and products (flavin-Gal*p* adduct) are presented in Table 1. It is observed that the cyclic oxygen, O5X_GAL_, losses considerable electron density in going from reactants to TS1, but it partially recovers it when the adduct is finally reached. C1X_GAL_, on the other hand, gains substantial electron density along the whole process. Finally, the partial charge of N5_FADH_ increases from −0.18 to 0.14 while its configuration changes from planar to tetrahedral. We note that the substantial loss of electron density of the nucleophile nitrogen in this step was predicted by the experiments in which FAD analogues with different electron-withdrawing/donating groups where used to determine the 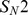 character of this step [46]. These changes weaken the N5_FADH_-H bond facilitating the transference of the proton during the next step. The evolution of the Cremer-Pople angles is shown in Fig. S1 panel b). At the Michaelis complex, the pyranose ring shows a ^4^C_1_ conformation and its 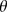 angle oscillates around 0^0^. However, after surmounting the transition state, this angle increases by ∼50^0^ while 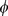 diminishes to -60^0^. This correspond to a ^2^H_3_ six membered ring conformation. This conformation persists until the formation of the flavin-galactose adduct, when the pyranose ring returns to ^4^C_1_.

**Table 1:** Partial charges of key atoms for the first two steps of the mechanism.

Atom	a	TS1	b	TS2	c	TS3	d
O3B_UDP_	−0.45(0.02)	−0.65(0.02)	−0.76(0.02)	−0.77(0.03)	−0.75(0.02)	−0.76(0.02)	−0.78(0.03)
C1X_GAL_	0.40(0.01)	0.31(0.02)	0.18(0.04)	0.26(0.01)	0.25(0.01)	0.01(0.03)	−0.07(0.05)
O5X_GAL_	−0.40(0.01)	−0.23(0.02)	−0.31(0.05)	−0.36(0.02)	−0.41(0.01)	−0.42(0.04)	−0.47(0.02)
N5_FADH_	−0.18(0.02)	−0.21(0.01)	0.05(0.03)	−0.11(0.02)	−0.13(0.02)	0.12(0.04)	0.20(0.03)
H_FADH_	0.25(0.01)	0.25(0.01)	0.24(0.01)	0.26(0.04)	0.37(0.01)	0.37(0.03)	0.33(0.02)
O4_FADH_	−0.72(0.02)	−0.69(0.02)	−0.43(0.02)	−0.61(0.03)	−0.44(0.02)	−0.57(0.04)	−0.66(0.03)
C4X_FADH_	−0.15(0.01)	−0.18(0.02)	−0.27(0.03)	−0.22(0.03)	−0.14(0.02)	−0.25(0.04)	−0.26(0.02)
C4_FADH_	0.36(0.021)	0.40(0.01)	0.33(0.02)	0.43(0.02)	0.33(0.01)	0.46(0.03)	0.48(0.04)
C5X_FADH_	0.10(0.01)	0.08(0.01)	0.00(0.02)	0.08(0.02)	0.09(0.01)	0.05(0.02)	0.02(0.03)

Charges are in atomic units. Labels a, b and c denote the species presented in Fig. 2. Numbers within parenthesis indicate the standard deviation of the charges.

**Figure 4:**
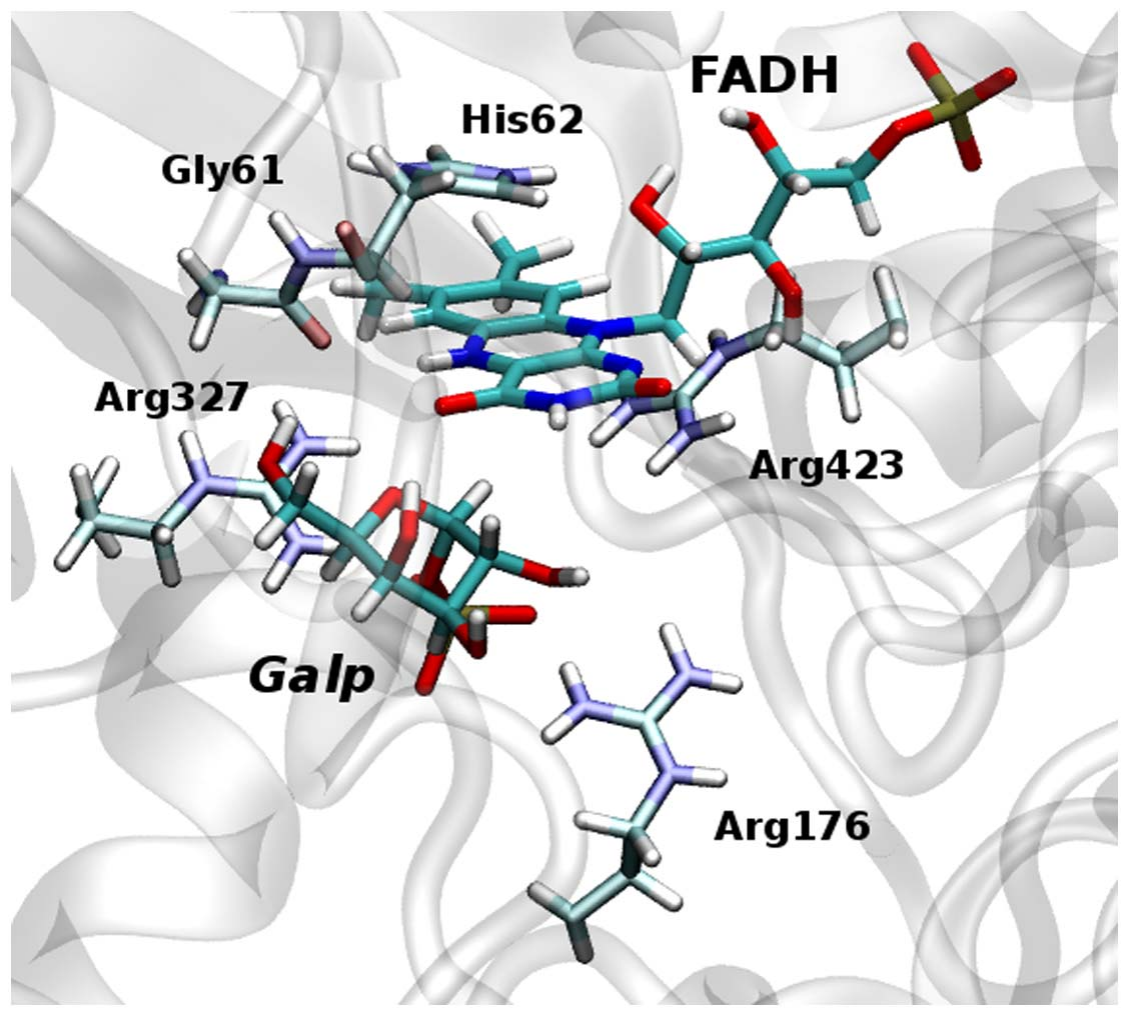
*Tc*UGM active site. Sketch of the active site of *Tc*UGM showing the relative positions of the FADH cofactor, the Gal*p* moiety of the substrate plus residues Gly61, His62, Arg176, Arg327 and Arg423.

The phosphate group of UDP bears strong H-bond interactions with Tyr395 and Tyr429 during the whole step. It also forms a H-bond with Arg327 but, at the Michaelis complex, this interaction is rather weak. However, once the covalent bond between UDP and the sugar is broken, the interaction gains strength because of the negative charge acquired by the reactive oxygen of the phosphate (see Table 1). Thus, while only 32.3% of the structures sampled before TS1 present a H-bond between Arg327 and the phosphate, the percentage raises to 69.7% for those sampled between TS1 and products. This indicates that Arg327 plays an important role in stabilizing TS1, as well as the products of the current step. Further support for this conclusion comes from Table 2 which shows that Arg327 has the most negative 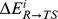 value. The location of this arginine within the active site can be seen in Figure 4. Arg327 is conserved in UGMs of both, prokaryotic and eukaryotic organisms [39]. For *Kp*UGM, it was found that its substitution by Ala completely abolishes the enzyme activity [48]. For *Tc*UGM the same substitution was found to reduce the catalytic activity, measured by 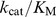, in 50% [29]. This effect is due to a reduction in 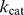 since the mutated enzyme has a larger affinity by the substrate, as indicated by the decrease in 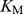 [29]. Our results agree with these experimental findings and suggest that the effect could be due to the role played by Arg327 in the catalysis of the flavin-galactose adduct formation. Finally, we note that the H-bonds between the phosphate and Tyr395, Tyr429 and Arg327 were found to be present in all of the remaining steps. Thus, they restrain the mobility of the phosphate group so that it is ready to re-bind the sugar moiety once the furanose ring is formed. This fact could explain the detrimental effect on 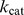 observed when Tyr395 and Tyr429 are substituted by Ala [29], and could also contribute to the negative effect observed upon the substitution of Arg327 with Ala.

**Table 2:** 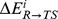
 values for key residues at every step of the reaction mechanism shown in Fig. 2.

Residue	ΔΔE step_1_	ΔΔE step_2_	ΔΔE step_3_	ΔΔE step_4_	ΔΔE step_5_	ΔΔE step_6_
	(kcal/mol)	(kcal/mol)	(kcal/mol)	(kcal/mol)	(kcal/mol)	(kcal/mol)
His62	0.74 (1.42)	−16.35 (0.77)	2.09 (1.13)	0.50 (0.90)	5.98 (1.54)	−3.37 (1.39)
Val63	0.01 (0.05)	−0.15 (0.04)	−0.05 (0.05)	−0.20 (0.05)	0.10 (0.04)	0.03 (0.05)
Arg176	−2.12 (0.80)	−1.76 (0.84)	3.47 (1.26)	1.06 (1.29)	−8.97 (2.04)	5.36 (1.24)
Asn201	−2.32 (0.45)	−1.37 (0.46)	0.12 (0.63)	1.58 (0.48)	−6.40 (0.61)	2.40 (0.94)
Tyr317	−0.19 (0.61)	3.18 (0.64)	−1.05 (0.63)	−0.49 (0.98)	−0.97 (0.54)	−0.82 (0.59)
Arg327	−16.15 (1.92)	4.72 (2.00)	−1.19 (1.75)	5.31 (1.82)	3.67 (2.39)	−8.10 (1.54)
Tyr395	−4.29 (0.68)	3.81 (0.51)	−0.41 (0.71)	−1.23 (1.04)	1.76 (0.84)	−2.27 (1.63)
Arg423	2.78 (0.65)	−3.18 (1.21)	5.01 (0.88)	2.86 (0.88)	6.53 (1.51)	1.00 (1.60)
Tyr429	−2.35 (0.65)	1.28 (0.93)	0.45 (0.96)	1.31 (1.06)	−1.15 (0.80)	0.60 (0.72)
Asn433	0.95 (0.42)	−1.26 (0.69)	−1.47 (0.41)	1.88 (0.58)	−1.23 (0.47)	−0.30 (0.87)

Numbers within parenthesis indicate the standard deviation of the energies.

Another interaction that is worth mentioning is the H-bond between the H atom bonded to N5_FADH_ and the carbonyl oxygen of Gly61. This interaction already exists at the reactants configuration but becomes stronger once TS1 is reached. The distance between the carbonyl oxygen of Gly61 and the H atom is 2.30 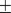 0.39 Å before TS1, but decreases to 1.89 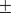 0.11 Å after it (see Fig. S1 panel b). Because of this, the H-bond is present in 26.4% of the configurations sampled before TS1 and 59.2% of those sampled afterwards. Since the interaction is stronger for TS1 than for reactants, it certainly helps to reduce the barrier to reaction. Unfortunately, the stabilizing effect of glycine residues cannot be evaluated with the energy decomposition method employed in this work. Because of this, Gly61 is not mentioned in Table 2. It has been found that the replacement of Gly61 with Ala or Pro has a profound detrimental effect on the activity of *Tc*UGM (∼90%) [38]. A putative explanation for this fact would be that these alternative residues reduce the flexibility of the backbone chain, hindering its ability to locate the carbonyl group in an appropriate position for the H-bond interaction. However, the evaluation of this hypothesis requires further MD computations that are outside the reach of this work.

### Stage 2: Formation of the iminium ion

This stage consists of two steps. The first one involves an intramolecular proton transfer from N5_FADH_ to O4_FADH_ (see Fig. 2, step 2). The calculated 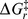 was 19.8 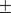 0.2 kcal/mol while 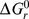 was −4.5 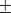 0.3 kcal/mol. The corresponding results of Huang *et. al.* for 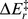 and 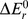 were 8.22 kcal/mol and −10.42 kcal/mol, respectively. Both calculations agree to indicate that the products of this step are more stable than the reactants. However, the energy difference is smaller in our computations and our estimated barrier is sensibly higher. The large barrier is not surprising since the initial distance between the H atom being transferred and the acceptor is rather long, 2.46 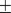 0.8 Å, while the donor-hydrogen-acceptor angle is far from collinear, 98.7 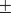 7.1 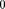. Because of this, a large configurational change needs to take place to enable the transference. The probability of H-tunneling for this step was found to be negligibly, with a transmission coefficient of 0.002 calculated at the zero point energy of the N-H bond. This results is not surprising since the barrier width is rather large.

In Fig. S2a we show the variations of the distances involved in the definition of the reaction coordinate 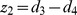 during step 2. At the transition state TS2 the distance between O4_FADH_ and the H atom has decreased by ∼1.3 Å but the N5_FADH_-H distance has not increased substantially. On the other hand the donor-acceptor distance, presented in panel (b), decreases by 0.36 Å in going from reactants to TS2. The analysis of the structures reveals that this shortening is mainly caused by the twisting of the C5X-N5-C4X-C4 torsion of FADH, also shown panel (b). This observation is confirmed by a correlation coefficient of 0.87 between the donor-acceptor distance and the corresponding dihedral angle. Thus, the ability of the isoalloxazine ring to bear this distortion contributes to make possible the transfer. In addition, Fig. S2b shows the distance between the H atom being transferred and O5X_GAL_. This distance reaches a minimum of 1.5 Å when the transference is complete generating a new H-bond between O4_FADH_ and O5X_GAL_. As can be seen in Table 1, the partial charge of O5X_GAL_ decreases along this step while that of the H atom increases. Both, the new H-bond and the variations in the partial charges make the scenario more prone to the attack of the proton onto O5X_GAL_ that initiates the ring opening at the following step. The partial charges of the N5_FADH_, C4X_FADH_, C5X_FADH_, C4_FADH_ and O4_FADH_ also suffer noticeable variations along this step. Basically, the donor and the acceptor of the proton gain electronic density in going from reactants to TS2, while C4X_FADH_, C5X_FADH_ and C4_FADH_ loss it.

Residue His62 lies pretty close to the isoalloxazine ring of FADH (See Fig. 4). They attract to each other since His62 has a positive charge while FADH has a negative charge. Along step 2 His62 moves closer to FADH and its side chain rotates so that the NE2 atom establishes a new H-bond with the hydroxyl group at position 2 of the ribitol chain of the cofactor. This movement strengthens the interaction between the two molecules. The importance of this variation can be appreciated from the values of 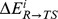 presented in Table 2. It is observed that His62 exerts the largest stabilizing effect on TS2, −16.35 kcal/mol, being the main responsible for the acceleration of this step. The involvement of His62 in the catalysis of step 2 could explain why the substitution of this residue by Ala diminishes the catalytic activity of *Tc*UGM in 98% [38].

The following step (step 3 of Fig. 2) involves the transfer of the H atom from O4_FADH_ to O5X_GAL_ to open the sugar ring and form the iminium ion. The calculated 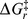 and 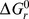 for this process are 13.7 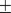 0.2 and −1.1 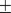 0.3 kcal/mol, respectively. Experimental results suggested that the opening of the ring, detected as the appearance of the iminium ion, is fast. [28] In agreement with the experimental evidence we found that this step presents a relatively small barrier. Huang *et. al.* estimated an energy barrier rather similar to our 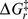, but their 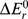 (8.12 kcal/mol) is quite distinct to our 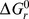 estimation. Fig. S3 shows the evolution of the distances involved in the definition of the reaction coordinate 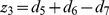. It is observed that at TS3 the proton is halfway between the donor and the acceptor, while the bond between O5X_GAL_ and C1X_GAL_ is partially broken. When this bond gets completely broken and the ring opens, the iminium ion is formed. During this process the distance between C1X_GAL_ and N5_FADH_ decreases from 1.50 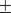 0.04 Å to 1.34 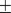 0.03 Å because the bond between them acquires a partial double bond character. In agreement with experimental findings [46], it was found that N5_FADH_ losses considerably electron density during this step (See Table 1). Besides two out the three atoms bound to this nitrogen (C1X_GAL_ and C4X_FADH_) gain electron density. The analysis of the TS3 stabilization pattern, presented in Table 2, shows that none of the active site residues has a significant influence on the energetic of this step. However, it should be kept in mind that the values of 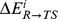 only provide information on static contributions to the interaction energy between a given residue and the quantum subsystem. Any effect of the residue on the conformational freedom of the active site will no be spotted by this analysis.

In that regard, it is interesting to analyse the movement of the hydroxyl groups of the sugar moiety once the chain is open. Fig. S4 shows the probability distributions for the dihedral angles C2X_GAL_-C3X_GAL_-C4X_GAL_-O4X_GAL_, C3X_GAL_-C4X_GAL_-C5X_GAL_-O5X_GAL_ and C4X_GAL_-C5X_GAL_-C6X_GAL_-O6X_GAL_. They describe the ability to rotate of the hydroxyl groups formed by O4X_GAL_, O5X_GAL_ and O6X_GAL_, respectively. The widest distribution corresponds to the hydroxyl group formed by O6X_GAL_, which rotates almost freely. On the contrary, the groups involving O4X_GAL_ and O5X_GAL_ liberate around to their average values. These two atoms participate in the bonds that close the ring in Gal*f* and Gal*p*, respectively. In both cases the impediment to rotate is mainly caused by the strong H-bonds that these hydroxyl groups maintain with O4_FADH_. The O4X_GAL_-O4_FADH_ H-bond is present in 95% of the configurations. The O5X_GAL_-O4_FADH_ H-bond is present in 96%. While the sugar chain remains open, these groups do not participate in any other hydrogen bonding interaction. The remaining hydroxyl groups of the sugar also form H-bonds. O3X_GAL_ interacts with Asn201 and O2X_GAL_ with the phosphate group.

### Stage 3: Formation of the flavin-Gal*f* adduct

This stage also has two steps. The first one is the cyclization of the sugar into the furanose form (step 4 of Fig. 2). It occurs accompanied of the proton transfer from O4X_GAL_ to O4_FADH_. We found that this step supports the highest barrier of the whole mechanism: 23.4 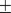 0.4 kcal/mol. This agrees with the measurements of Sobrado *et. al.* who determined that this formation of the furanose ring is sensible slower than the opening process *Tc*UGM [28]. The free energy change of the step is 2.9 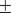 0.2 kcal/mol.

In Fig. S5 panel a) we show the evolution of the distances involved in the definition of the reaction coordinate 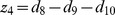, while panel (b) displays the dihedral angles that determine the orientation of the hydroxyl groups at positions 4 and 5. It is observed that, at the beginning of the process, the two hydroxyl groups change their orientation in a concerted way, while O4X_GAL_ and C1X_GAL_ approximate to each other. These movements destroy the H-bond interaction between O5X_GAL_ and O4_FADH_ and, initially, also drive the H atom to be transferred away from O4_FADH_. However, once the O4X_GAL_-C1X_GAL_ distance gets short enough, a fine tuning in the orientation of the hydroxyl group at position 4 is observed. This reorientation takes its H atom closer to O4_FADH_. Initially, while all these rearrangements take place, the H-O4X_GAL_ bond hardly stretches. Only when the O4X_GAL_-C1X_GAL_ distance gets smaller than 2.3 Å, the H-O4X_GAL_ bond starts to weaken. At the transition state, which appears rather late, the H-O4X_GAL_ and H-O4_FADH_ distances are almost the same. Finally, when the products configuration is reached and the sugar is in the furanose form, O5X_GAL_ and O6X_GAL_ present no H-bond interactions. In contrast, O3X_GAL_ and O2X_GAL_ keep their interactions with Asn201 and the phosphate group, respectively.

The stabilization pattern presented in Table 2 shows that none of the active site residues lowers the energy of TS4 with respect to the reactants in a significant amount. However we note that entropy could play an important role for this step. In general, the conformational freedom of a sugar moiety is larger when the chain is open than when it is closed. Accordingly, it is expected that the cyclization process occurs accompanied by a continuous loss of entropy in the sugar molecule. This would make the reaction slower and the equilibrium position more favourable to the open form. In water solution this effect is compensated by an increase in the entropy of the solvent, but the situation is different within an enzyme. The results of the MD simulation for the open form, discussed at the end of the previous section, suggest that for the reaction under analysis this deleterious entropic effect is ameliorated by the interactions between the carbonyl oxygen of the cofactor and the -OH groups at positions 4 and 5 of the sugar moiety. These interactions attenuate the mobility of the open form and therefore reduce its entropy. Consequently, the entropy change in going from reactants to TS4 or to products is not so adverse. We note, however, that our MD runs are not long enough to allow for an accurate estimation of entropic effects. Thus, the hypothesis put forth in this paragraph needs to be evaluated by additional simulations, especially tailored to that end.

A direct comparison with the results of Huang *et. al.* cannot be done for this step because the authors split the process in three parts. First, a rotation around the C4X_GAL_-C5X_GAL_ bond to take O5X_GAL_ away from O4_FADH_; second a rotation around the C4X_GAL_-C3X_GAL_ bond to place O4X_GAL_ close to C1X_GAL_; third, the attack of O4X_GAL_ onto C1X_GAL_. We found that the computed free energy curve along reaction coordinate 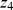 presents no intermediate minimum, indicating that the ring closure takes place in a single concerted step. In other words, the energy minima corresponding to the intermediates detected by Huang *et. al.* do not appear in our free energy computations. This difference could be attributed to dynamical effects which, as explained above, are expected to be large for this cyclization process but are not considered in electronic structure computations.

In the second step of this stage (step 5 of Fig. 2) the H atom is transferred back from O4_FADH_ to N5_FADH_. The calculated 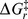 and 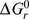 for this process are 19.8 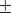 0.4 and 1.0 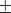 0.3 kcal/mol, respectively. These results are similar to those of Huang *et. al*. It should also be noted that the barrier and free energy change of this step are pretty similar to those of the reverse of step 2. The result is not surprising since the only difference between them is whether the sugar moiety is in the furanose or pyranose form. The transmission coefficient for H-tunneling for this step is more than fifteen times smaller than that of step 2. This is due to the fact that this step is slightly endothermic while step 2 is exothermic. Nevertheless, in both cases, the probability for H-tunneling is negligibly.

In Fig. S6a we present the evolution of the distances involved in the definition of reaction coordinate 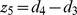, while in panel (b) we show the evolution of the C1X_GAL_-N5_FADH_ distance and the Cremer-Pople angle 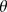 of the furanose ring. The curves for the H-O4_FADH_ and H-N5_FADH_ distances evolve according to the expectations for a direct proton transfer. However, the curve of the C1X_GAL_-N5_FADH_ distance shows an unexpected increase after TS5. The final value, ∼1.85 Å, is significantly larger than a typical C-N single bond. Our results thus indicate that the adduct between Gal*f* and FADH becomes rather weak when N5_FADH_ adopts the sp3 hybridization. The enlargement of the C1X_GAL_-N5_FADH_ distance was also described by Huang *et. al*. However, it that case, the final value was somewhat smaller than in our calculations (1.70 Å). In order to check the final distance between C1X_GAL_ and N5_FADH_ we re-simulated the transference using longer simulation times for each window, as well as employing larger QM subsystems. However, we consistently obtained the same result. Moreover, the H transference was simulated applying a restriction on the C1X_GAL_-N5_FADH_ distance, so that it was forced to get values smaller than 1.65 Å. These calculations provided higher 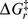 and 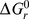 than those obtained without the restriction. Besides, when an unrestricted MD was performed on the products of the restricted transfer, the system spontaneously relaxed to a stable conformation with a C1X_GAL_-N5_FADH_ distance of ∼1.85 Å. Fig. S6b indicates that the increase in the C1X_GAL_-N5_FADH_ distance is accompanied with an increase in the Cremer-Pople angle 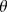. This takes the configuration of the sugar ring from 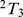, for reactants, to 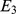 for products. It has to be noted that both, the enlargement of the C1X_GAL_-N5_FADH_ distance and the change in the conformation of the furanose ring, are required to avoid the steric clash between the cofactor and the substrate. The values of 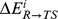 presented in Table 2 show that His62 and Arg423 destabilize TS5, but the effect is more than compensated by the stabilization produced by Arg176 and Asn201. Site directed mutagenesis experiments determined that the mutation of Arg176 by Ala produces an impressive reduction in 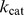 [29]. The involvement of this residue in the catalysis of step 5 could be one of the reasons of this finding.

### Stage 4: Formation of UDP-Gal*f*

At first sight, this stage could be considered as the reverse of stage 1, except for the fact that the sugar is now in the funarose form. However, as stated in the previous section, the flavin-Gal*f* bond is already very weak when the transference of the proton from O4_FADH_ to N5_FADH_ is completed. Because of this, the barrier for this step is pretty low, 5.8 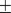 0.2 kcal/mol, and 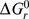 is quite negative, −14.8 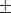 0.1 kcal/mol. In Fig. S7 we show the evolution of the distances involved in the definition of reaction coordinate 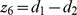. At the transition state, the N5_FADH_ -C1X_GAL_ and C1X_GAL_-O3B_UDP_ distances are both 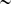 2.18 Å. This corresponds to a bond order of ∼0.32 for the two bonds. As in the case of the flavin-Gal*p* adduct formation, this is consistent with a dissociative S*_N_*2 mechanism. The evolution of the Cremer-Pople angle 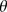 of the furanose ring is also shown in Fig. S7. It changes from 255^0^ to 240^0^, indicating that the ring conformation returns from 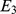 to 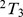. Once the products are formed, the O5X_GAL_ hydroxyl group establishes a new H-bond with the phosphate group. Table 2 shows that the stabilization pattern of this step is similar to that of step 1, with Arg327 being the most stabilizing residue of the TS.

## Conclusions

We have presented a detailed description of the energetic and structural changes that take place during the entire mechanism of the reaction catalysed by *Tc*UGM. The results confirm and explain several previous experimental findings, and they also provide new insights on the dynamics of the active site along the reaction.

In agreement with experiments, our results confirm that the first stage of the reaction (formation of the Gal*p*-flavin adduct) proceeds on a single step via a S*_N_*2 dissociative mechanism [28], [ 34], [ 46]. Moreover, the computations indicate that Arg327 is the main responsible for the selective stabilization of the TS of this step. This could explain why the substitution of this Arg by Ala reduces the 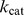 of *Tc*UGM by 69% [29]. The carbonyl oxygen of Gly61 also plays a role in that regard. However, the stabilization energy of this residue could not be quantified.

The second stage of the reaction (formation of the iminium ion) occurs in two steps, as predicted by the electronic structure calculations of Huang *et. al*. First, a proton passes from N5_FADH_ to O4_FADH_. This is followed by the transfer of the proton from O4_FADH_ to O5X_GAL_ that triggers the opening of the ring. The energy decomposition analysis indicated that residue His62 is the main responsible for the catalysis of the first transference. This result could explain why mutations introduced on that position produced a high detrimental effect on the activity of *Tc*UGM. The transfer of the proton from O4_FADH_ to the cyclic oxygen, on the other hand, presents a relatively low barrier and none of the active site residues is particularly relevant to stabilize or destabilize its TS with respect to the reactants.

At the end of stage 2, galactose is in the open-chain form. We analysed, the interactions and dynamics of the hydroxyl groups of the sugar in such situation. We found that the group at position 6 moves freely, without interacting with any active site residue, the one at position 3 forms a H-bond with Asn201 while the one at position 2 forms a H-bond with the phosphate group of UDP. More importantly, the hydroxyl groups at positions 4 and 5 strongly interact with O4_FADH_ via H-bonds. These interactions significantly reduce the conformational freedom of the sugar moiety.

During the closing of the sugar ring to form Gal*f*, the dihedral angles around the C2X_GAL_-C3X_GAL_ and C3X_GAL_-C4X_GAL_ bonds rotate in a concerted way. These rotations take O5X_GAL_ away from O4_FADH_ and locate O4X_GAL_ close to C1X_GAL_. It was found that this step presents the highest free energy barrier of the whole mechanism, in agreement with the proposal of Sobrado *et. al.* [28]. The energy decomposition analysis showed that none of the active site residues is particularly important to reduce the energy of the TS with respect to the reactants, but the MD results discussed in the previous paragraph suggested that entropy could play an important role. In general, the cyclization of a sugar chain occurs with a reduction of its entropy, a fact that hampers the reaction. In this case, by reducing the conformational freedom of the open-chain form, the active site of *Tc*UGM could make the entropy change and the activation entropy of this step less adverse. Unfortunately, the characteristics of our simulations do not allow to quantify this effect. We note, however, that since this step has the largest free energy barrier, any small reduction on that barrier can be significant.

Once Gal*f* is formed, the next step involves the transference of the proton bound to O4_FADH_ towards N5_FADH_. We observed that something unexpected occurs during this process. Once the system has passed over the TS, the furanose ring changes its conformation from 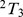 to 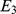 while the distance between C1X_GAL_ and N5_FADH_ increases to get a final value of ∼1.85 Å. The visual inspection of the structures reveals that these modifications are required to avoid the steric clash between the substrate and the cofactor. Huang *et. al.*, who used a different level of theory, different quantum subsystem and different model for the active site, also found a rather long C1X_GAL_-N5_FADH_ distance at the end of this transference. Residues Arg176 and Asn201 make the main contributions to the lowering of the barrier. This role of Arg176 is in line with recent experiments which found that the mutation of this residue by Ala reduce the 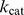 of *Tc*UGM [29]. During the last step of the reaction, the sugar in the furanose form re-binds to UDP as it detaches from the cofactor. Since the C1X_GAL_-N5_FADH_ bond is already rather weak at the end of the previous step, this last transformation presents a small barrier and a very negative energy change.

Tyr395 and Tyr429 also play an important role in the reaction. Both residues bear strong H-bond interactions with the phosphate group of the cofactor. These bonds are stable throughout the whole catalysed mechanism. Since these interactions are always present, they do not modify the energy of the barriers found along the reaction. Instead, they facilitate the process by keeping the phosphate group at a relatively fixed position, close to the sugar moiety. Thus, UDP is ready to re-bind to the sugar once it adopts the furanose form. Not surprisingly, experiments determined that the substitution of any of these tyrosines by phenylalanine reduced the 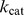 of *Tc*UGM [29].

Summarizing, the QM/MM molecular dynamics computations presented in this article determined that residues His62, Arg176, Asn201 and Arg327 contribute to the catalytic activity of *Tc*UGM by reducing the barriers of different steps of the mechanism. Tyr385 and Tyr429, on the other hand, play a role by keeping UDP always close to the sugar moiety. Also, the results highlight the participation of the carbonylic oxygen at position 4 of the cofactor. As predicted by Huang *et. al.* this atom provides an alternative route for the transference of the proton between N5_FADH_ and the cyclic oxygen of the substrate. Without this route the barrier for the transference would be prohibitively high. Besides this oxygen restricts the mobility of the open-chain form of the sugar facilitating the ciclyzation process. We hope that the insights obtained from this computational study can contribute to the design of efficient inhibitors of *Tc*UGM.

## Methods

### Initial settings

The crystallographic structure of reduced *Tc*UGM with UDP was taken from the Protein Data Bank, entry 4DSH. To determine the coordinates of Gal*p* within UGM we superimposed the UDP-Gal*p* molecule, taken from the crystal structure of *Asparragilus fumigatus* UGM (PDB code 3UKF), with the crystallographic UDP of *Tc*UGM. The resultant coordinates of UDP-Gal*p*, together with those of *Tc*UGM, were used as the starting geometry of *Tc*UGM in its holo form. In the initial configuration the nucleophilic group and the leaving group laid on opposite sides of the sugar ring. The distance between C1X_GAL_ and N5_FADH_ was 3.78 Å. The angle between N5_FADH_, C1X_GAL_ and the oxygen atom of UDP, O3B_UDP_, was 144.2°. The flavin cofactor was set in the reduced deprotonated state since it was recently shown that this form augments the nucleophilic character of N5_FADH_ [40]. Besides, since experiments indicate that the pKa of N1_FADH_ is ∼ 6.7 while that of N5_FADH_ is 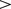 20, the proton of the reduced flavin was located on N5_FADH_ [49]. The protonation state of the enzyme residues was assigned according with the standard rules except for His62, since recent experiments showed that this residue is protonated when the cofactor is in the reduced state [38]. The resulting file was fed into the Leap module of AMBER and the system was solvated in a 10.0 Å truncated octahedral cell of TIP3P explicit water molecules [50], including the crystallographic water molecules.

The QM/MM molecular dynamics and free energy simulations were performed with the AMBER12 package [51], using periodic boundary conditions with a cutoff distance of 10.0 Å and a time step of 1.0 fs. The potential energy of the classical region was computed with the Amber99SB force field [52] while the self-consistent charge Density Functional Tight Binding method (scc-DFTB) [51] was employed for the QM subsystem. The DFTB method has proved to be appropriate to describe the energetics of many chemical [53] and biochemical reactions [54]–[56]. More recently, it was shown to provide the best semiempirical description for six-membered carbohydrate rings deformation [57], [ 58]. The QM subsystem was formed with the flavin cofactor, the substrate, Gly61, His62, Val63, as well as the lateral chains of Arg176, Arg327 and Arg423. This adds up to 232 atoms with a net charge of -1.

The initial structure was first minimized at constant volume and then heated at NVT conditions from 0 K to 310 K by a simulated annealing technique. A weak harmonic restraint on the C*_α_* atoms was implemented during this period. This was followed by 200 ps of equilibration at NPT conditions at 310 K and 1 bar. No restrains were applied in this case. The Pauling Bond Orders, 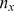, were determined when galactose either attaches or detaches from the flavin cofactor (process a
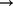
b and f
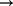
g of Fig. 2). In both cases, the bonds involved are C-O and C-N. The equation used to calculate the orders was, 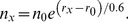
(1)


Here 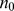 denotes the bond order of the fully formed bond while 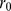 is the equilibrium distance, which was considered equal to 1.5 Å for the two bonds involved in these reactions. The value of 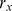 was computed as the average distance among the structures sampled in the umbrella simulations at the transition state. The presence of H-bonds was monitored considering that a H-bond exists if the distance between the donor and the acceptor is 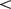 3.15 Å and the donor-H-acceptor angle is 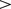 145°.

When relevant (steps 2 and 5), the probability of H-tunneling was estimated employing the expression for the microcanonical transmission coefficient given at equation 14a of reference [59]. This expression corresponds to tunneling through a one-dimensional barrier whose shape, height and exothermicity are determined by three adjustable parameters. In our estimations these parameters were obtained by fitting the free energy curves of the corresponding proton transfer steps. Since the coordinate in these curves is not the proton coordinate but a difference between the distances of the bond being broken and formed, the effective mass of the particle being transferred was set as 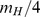, where 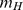 is the mass of the proton [60]. The energies employed in these estimations were 1700 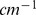 for step 2 and 1800 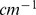 for step 5. These are approximately the zero point energies of the N-H and O-H bonds.

### Umbrella sampling calculations

The umbrella sampling technique was employed to analyse all the steps involved in the conversion between Gal*p* and Gal*f* within *Tc*UGM (see Fig. 2). Free energy profiles were computed along different reaction coordinates, conveniently defined for each transformation. Harmonic restraints were applied in order to force the system to wander around the selected values of the reaction coordinate. A restraining force of 350.0 kcal/molÅ^2^ was employed in all cases and the reaction coordinate was sampled considering windows of 0.08 Å wide. Within each window, an equilibration phase of 65 ps was followed by a production phase of 0.2 ns. The actual values of the reaction coordinate were recorded every 2 fs. Snapshots of the structures were downloaded every 3 ps. The last 30000 data of each window were used to compute the unbiased probability by means of the weighted histogram analysis method (WHAM) [61]. When following each reaction coordinate, the last structure of a given window was used as the starting point for the next one. Simulations of 0.5 ns without any restraint were performed for reactants, products and for each intermediate species in order to check their stability. To check the convergence of the free energy computations, several tests were performed. First, for each step, we compared the free energy profiles obtained using the first half of the data (i.e. the first 15000 values selected at each specific value of the reaction coordinate) with the second half. Besides, every reaction coordinate was sampled both, forward and backwards. Finally, computations for steps 3 and 4 (the opening and closure of the sugar ring) were repeated three times using different initial configurations. Summing all the steps of the reaction, with their corresponding convergence tests, the total length of the QMMM-MD simulations was 187.5 ns. Below we provide the numerical details of the umbrella sampling calculations for each stage.

#### Stage 1: Formation of the flavin-Gal*p* adduct

This stage consists of just one concerted step in which the bond between Gal*p* and UDP breaks while Gal*p* joins FADH^−^ (step 1 in Fig. 2). Accordingly, the reaction coordinate was defined as 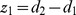, where 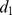 is the distance between C1X_GAL_ and N5_FADH_, while 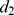 is the distance between C1X_GAL_ and O3B_UDP_. This coordinate was sampled from −2.03 to 1.89 Å.

#### Stage 2: Formation of the iminium ion

This stage was proposed to occur in two consecutive steps. The first one is the tautomerization of FADH^–-^ via the transfer of the H atom bonded to N5_FADH_ towards O4_FADH_ (step 2 in Fig. 2). The reaction coordinate for this tautomerization was defined as 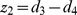, where 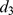 denotes the N5_FADH_-H distance while 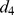 is the O4_FADH_-H distance. Coordinate 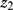 was sampled from −1.63 to 1.73 Å.

During the following step, the opening of the ring is initiated by the transfer of the proton linked to O4_FADH_ towards the oxygen atom of the Gal*p* ring, O5X_GAL_, and proceeds with the breakage of the bond between C1X_GAL_ and O5X_GAL_ (step 3 in Fig. 2). We found that a correct description of this process required a reaction coordinate defined as 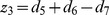, where 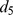 is the distance between O4_FADH_ and the proton being transferred, 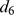 is the distance between C1X_GAL_ and O5X_GAL_ and 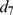 is the distance between the proton and O5X_GAL_. Coordinate 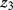 was sampled from 0.82 to 4.18 Å.

We also analysed the possibility of a direct proton transfer from N5_FADH_ to O5X_GAL_. In order to do that we defined a reaction coordinate 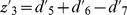, where 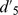 is the N5_FADH_-H distance, 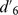 the one between O5X_GAL_ and C1X_GAL_ and 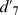 the O5X_GAL_-H distance. This coordinate was sampled from −1.85 to 0.71 Å. In agreement with previous results of Huang *et. al.* [41] we found that this direct proton transfer is very unlikely since it has a barrier significantly higher than the alternative path.

#### Stage 3: Formation of the flavin-Gal*f* adduct

This stage also occurs in two steps. First, the hydrogen attached to O4X_GAL_ is transferred to O4_FADH_ while a bond between O4X_GAL_ and C1X_GAL_ is formed (step 4 in Fig. 2). The reaction coordinate for this step was defined as 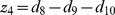, where 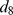 is the distance between the H atom being transferred and O4X_GAL_, 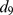 the distance between the H atom and O4_FADH_ and 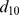 is the distance between O4X_GAL_ and the C1X_GAL_. Coordinate 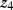 was sampled from −4.85 to −1.01 Å.

The following step consists of a proton transfer from O4_FADH_ to N5_FADH_ (step 5 in Fig. 2). This can be seen as the reverse of step 2, except for the fact that galactose is now in the furanose form. Therefore, the reaction coordinate was defined as the reverse of step 2 (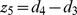) and it was scanned from −1.63 to 1.65 Å.

#### Stage 4: Formation of UDP-Gal*f*

This last step corresponds to the breakage of the bond between FADH^−^ and Gal*f* along with the formation of a bond between Gal*f* and UDP (step 6 of Fig. 2). Since this process is analogous to step 1 but occurs in reverse sense we defined 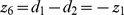 and scanned it from −1.98 to 1.38 Å.

### Energy decomposition

An energy decomposition analysis was performed to evaluate how the active site residues stabilize or destabilize the transition states of the successive steps with respect to their correspondent reactants. Different variations of this idea have been implemented to study enzymatic reactions [62]–[72]. In this case we followed the approach recently employed to compare the catalytic mechanisms of *T. cruzi* transialidase and *T. rangeli* sialidase [54]. Since the approach has been discussed in detail elsewhere we only present here the most relevant equations.

In the QM/MM study of an enzymatic reaction the influence of the classical environment on the activation energy of a given step, 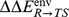, can be evaluated as, 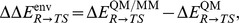


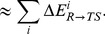
(2)


Here 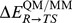 is the activation energy within the enzyme (computed with the QM/MM approach) while 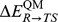 is the activation energy of the isolated quantum subsystem (computed at the same QM level). The terms appearing in the summation, 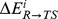, measure the influence of each individual residue on the reaction barrier. They are strictly given by, 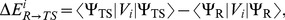
(3)where 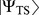 and 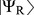 are the wave functions of the quantum subsystem at the transition state and reactants configurations, respectively, while 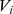 is the non-bonded interaction energy of classical residue 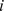 with the quantum subsystem. The evaluation of 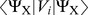, with X = TS or R, is not trivial since the AMBER code does not compute these values. Instead it provides the energy of the whole system, which accounts for the quantum hamiltonian, 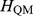, plus the sum of all the non-bonded interactions between the QM subsystem and the classical environment. Thus we estimated each 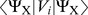 as, 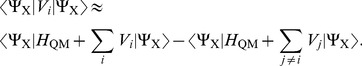
(4)


Here the first term on the right side gives the actual energy of the system at the given configuration. The second one is a fictitious energy calculated with the same wave function by setting the classical environment at exactly the same configuration except for the 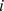-th residue which is transformed into Gly. Average values of 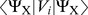, with X = TS or R, were computed employing 100 snapshots taken from the umbrella sampling calculations with the reaction coordinate set at the TS or reactants configurations, respectively. For these calculations we defined the QM subsystem as the substrate plus the cofactor, while the active site residues under analysis were His62, Val63, Arg176, Asn201, Tyr317, Arg327, Tyr395, Arg423, Tyr429 and Asn433.

The 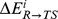 computed in this way measures the difference between the actual barrier to reaction and the barrier that would be observed if the interaction between the side chain of residue 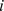 and the QM subsystem were turned off. Because of this, neither can it provide information about the effect of the backbone atoms or the effect of Gly residues. Moreover, since no dynamics is run when the 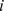-th residue is replaced by Gly, 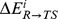 does not take into account dynamic effects arising from changes in the conformational freedom of the enzyme upon replacement. Finally we note that positive/negative values of 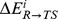 provide a strong indication about a deleterious/beneficial effect of residue 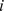 for the reaction step under consideration. However, they cannot be used to quantitatively estimate changes in 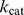 produced by the mutation of the 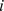-th residue by Gly because such changes depend on variations in the activation free energy, 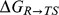.

## Supporting Information

Figure S1
**Evolution of important distances and angles along step 1.** Panel (a): evolution of the distances involved in the definition of the reaction coordinate 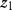. Panel (b): evolution of the Cremer-Pople angles 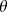 and 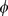 along with the distance between the H atom bonded to N5_FADH_ the carbonyl oxygen of Gly61. The location of the transition state is indicated with an arrow.(TIFF)

Figure S2
**Evolution of important distances and angles along step 2.** Panel (a): evolution of the distances involved in the definition of the reaction coordinate 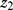. Panel (b): evolution of the distance between the donor and the acceptor of the proton, the distance between the proton and the cyclic oxygen, and the torsional angle defined by C5X-N5-C4X-C4 of FADH. The location of the transition state is indicated with an arrow.(TIFF)

Figure S3
**Evolution of important distances along step 3.** Evolution of the distances involved in the definition of the reaction coordinate 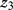. The location of the transition state is indicated with an arrow.(TIFF)

Figure S4 **Probability distributions of the torsional angles defining the orientation of the hydroxyl groups at positions 4, 5 and 6 of the sugar moiety in the open form.** The bars of the histogram are scaled so that the most likely angles of each distribution have a unitary height.
(TIFF)

Figure S5
**Evolution of important distances and angles along step 4.** Panel (a): evolution of the distances involved in the definition of the reaction coordinate 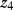. Panel (b): torsional angles that define the orientation of the hydroxyl groups at positions 4 and 5 of galactose. The location of the transition state is indicated with an arrow.(TIFF)

Figure S6
**Evolution of important distances and angles along step 5.** Panel (a) evolution of the distances involved in the definition of the reaction coordinate 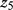. Panel (b) distance between N5_FADH_ and C1X_GAL_ along with the 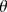 Cremer-Pople angle of furanose. The location of the transition state is indicated with an arrow.(TIFF)

Figure S7
**Evolution of important distances and angles along step 6.** Evolution of the distances involved in the definition of the reaction coordinate 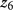 along with the 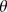 Cremer-Pople angle of the furanose ring. The location of the transition state is indicated with an arrow.(TIFF)

Text S1 **PDB file for UDP-Gal** ***p*** **bound to UGM (Michaelis complex).** This species is labelled as a in Fig. 2.
(PDB)

Text S2 **PDB file for the flavin-Gal** ***p*** **adduct in UGM.** This species if labelled as b in Fig. 2.
(PDB)

Text S3 **PDB file for the third species of the mechanism proposed for the reaction catalysed by UGM.** This species is labelled as c in Fig. 2.
(PDB)

Text S4 **PDB file for the iminium ion in UGM.** This species is labelled as d in Fig. 2.
(PDB)

Text S5 **PDB file for the fifth species of the mechanism proposed for the reaction catalysed by UGM.** This species is labelled as e in Fig. 2.
(PDB)

Text S6 **PDB file for the flavin-Gal** ***f*** **adduct in UGM.** This species is labelled as f in Fig. 2.
(PDB)

Text S7 **PDB file for UDP-Gal** ***f*** **bound to UGM.** This species is labelled as g in Fig. 2.
(PDB)
