# Supplementary figures and images for: QM/MM Molecular Dynamics Study of the Galactopyranose → Galactofuranose Reaction Catalysed by Trypanosoma cruzi UDP-Galactopyranose Mutase
Source: PLoS One. 2014 Oct 9;9(10):e109559. doi: 10.1371/journal.pone.0109559 (PMC4192007; doi:10.1371/journal.pone.0109559)

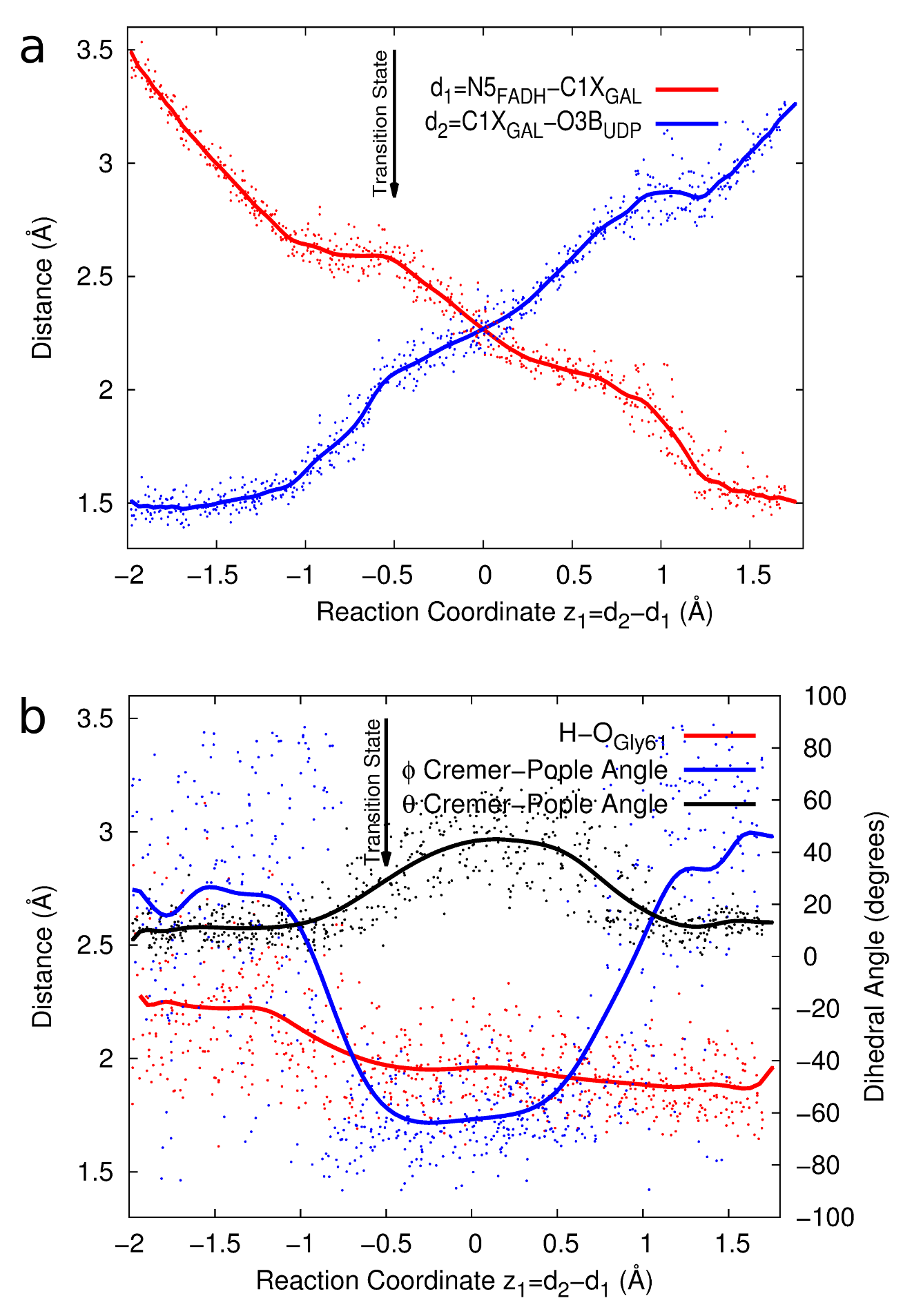

Supplement: Figure S1 — Evolution of important distances and angles along step 1. Panel (a): evolution of the distances involved in the definition of the reaction coordinate . Panel (b): evolution of the Cremer-Pople angles and along with the distance between the H atom bonded to N5FADH the carbonyl oxygen of Gly61. The location of the transition state is indicated with an arrow. (TIFF) [file pone.0109559.s001.tiff]

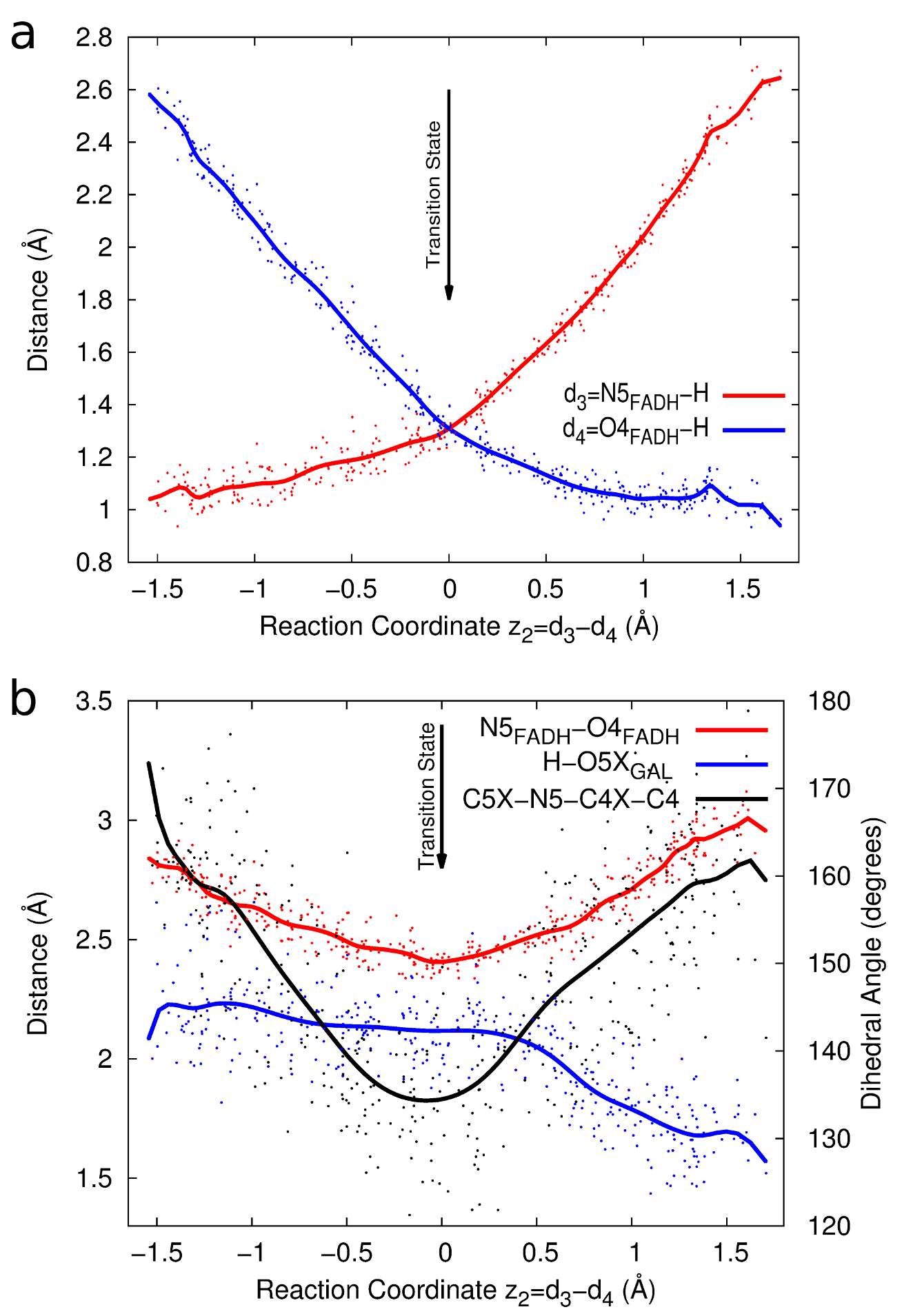

Supplement: Figure S2 — Evolution of important distances and angles along step 2. Panel (a): evolution of the distances involved in the definition of the reaction coordinate . Panel (b): evolution of the distance between the donor and the acceptor of the proton, the distance between the proton and the cyclic oxygen, and the torsional angle defined by C5X-N5-C4X-C4 of FADH. The location of the transition state is indicated with an arrow. (TIFF) [file pone.0109559.s002.tiff]

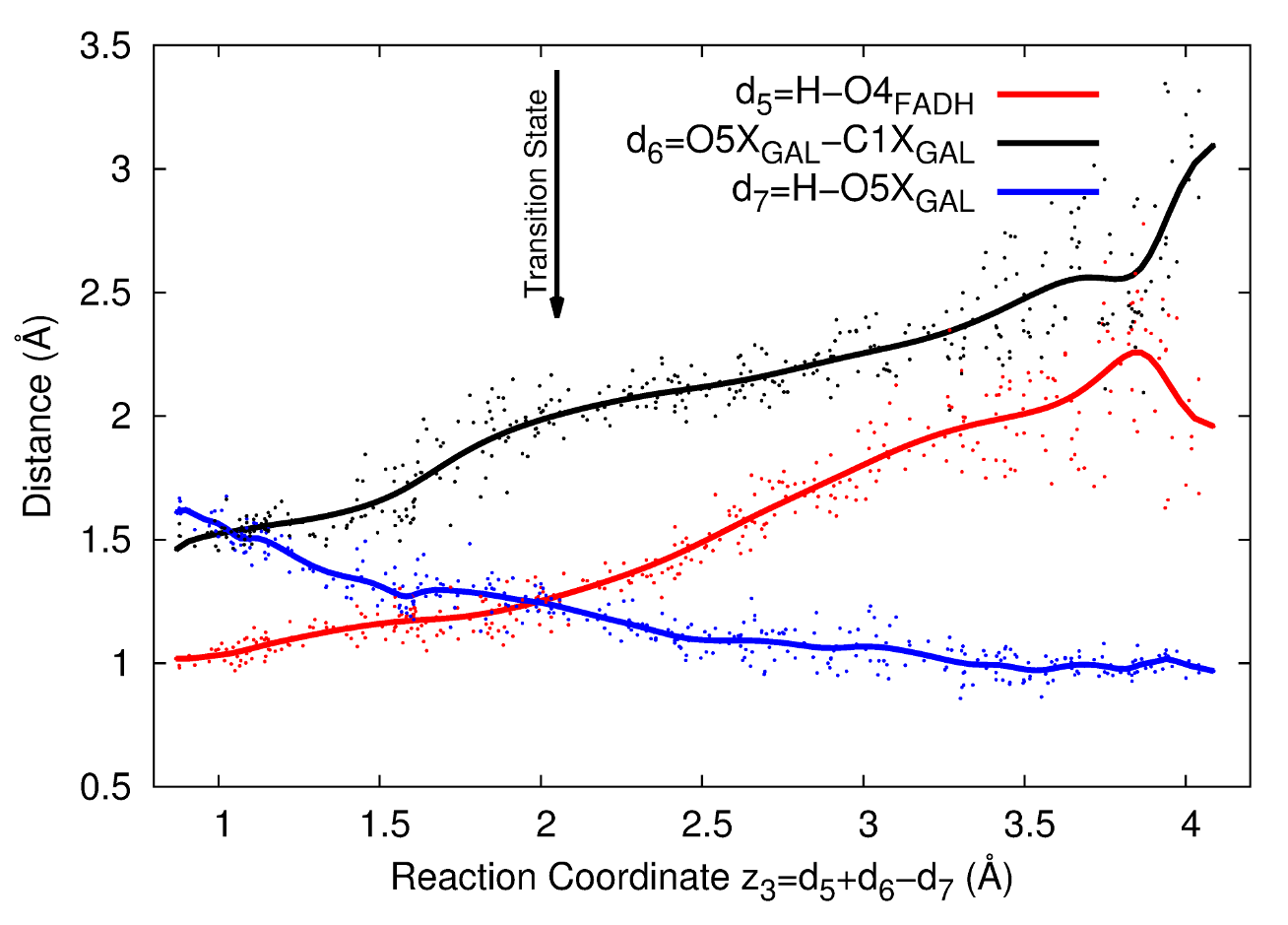

Supplement: Figure S3 — Evolution of important distances along step 3. Evolution of the distances involved in the definition of the reaction coordinate . The location of the transition state is indicated with an arrow. (TIFF) [file pone.0109559.s003.tiff]

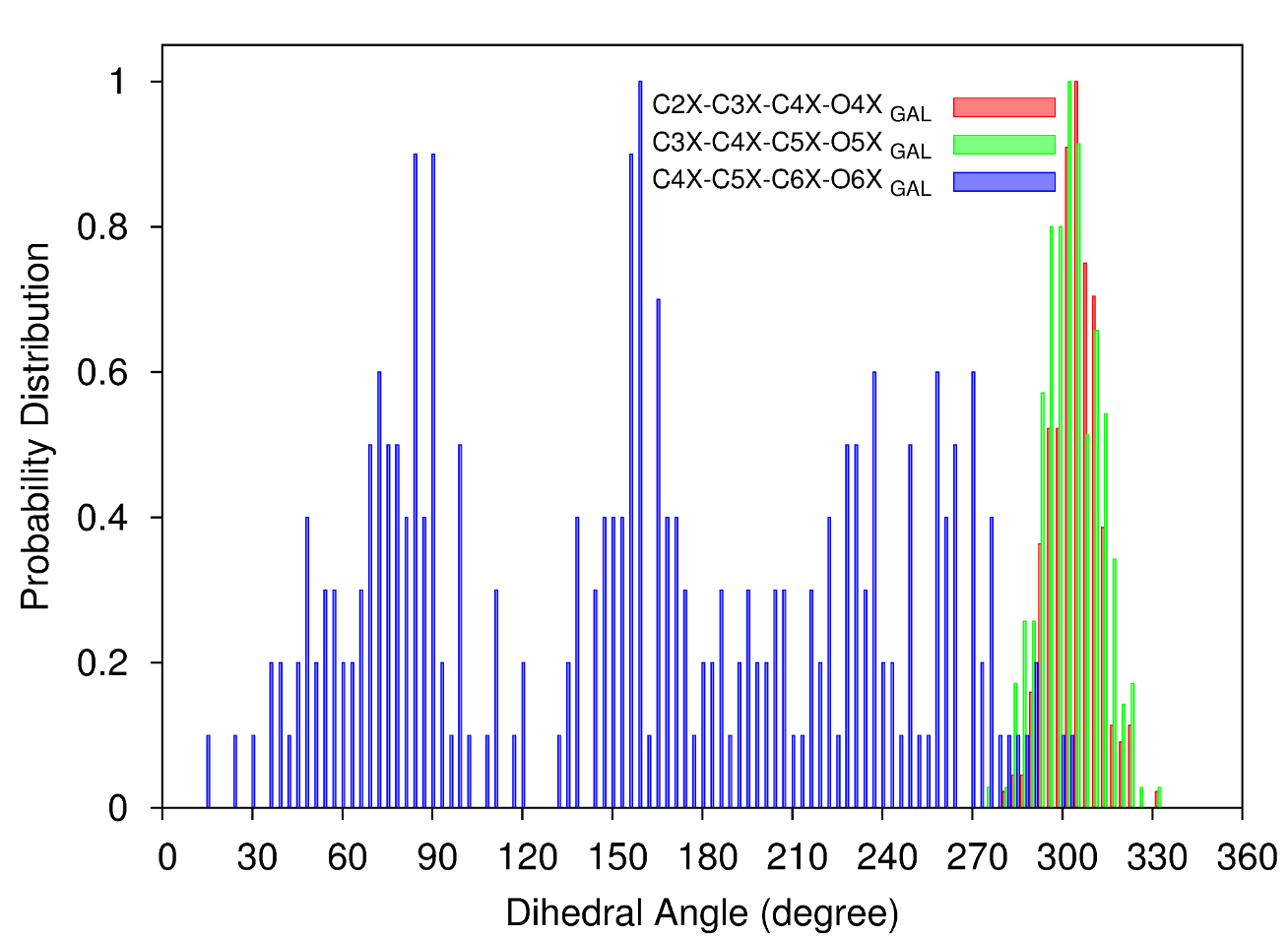

Supplement: Figure S4 — Probability distributions of the torsional angles defining the orientation of the hydroxyl groups at positions 4, 5 and 6 of the sugar moiety in the open form. The bars of the histogram are scaled so that the most likely angles of each distribution have a unitary height. (TIFF) [file pone.0109559.s004.tiff]

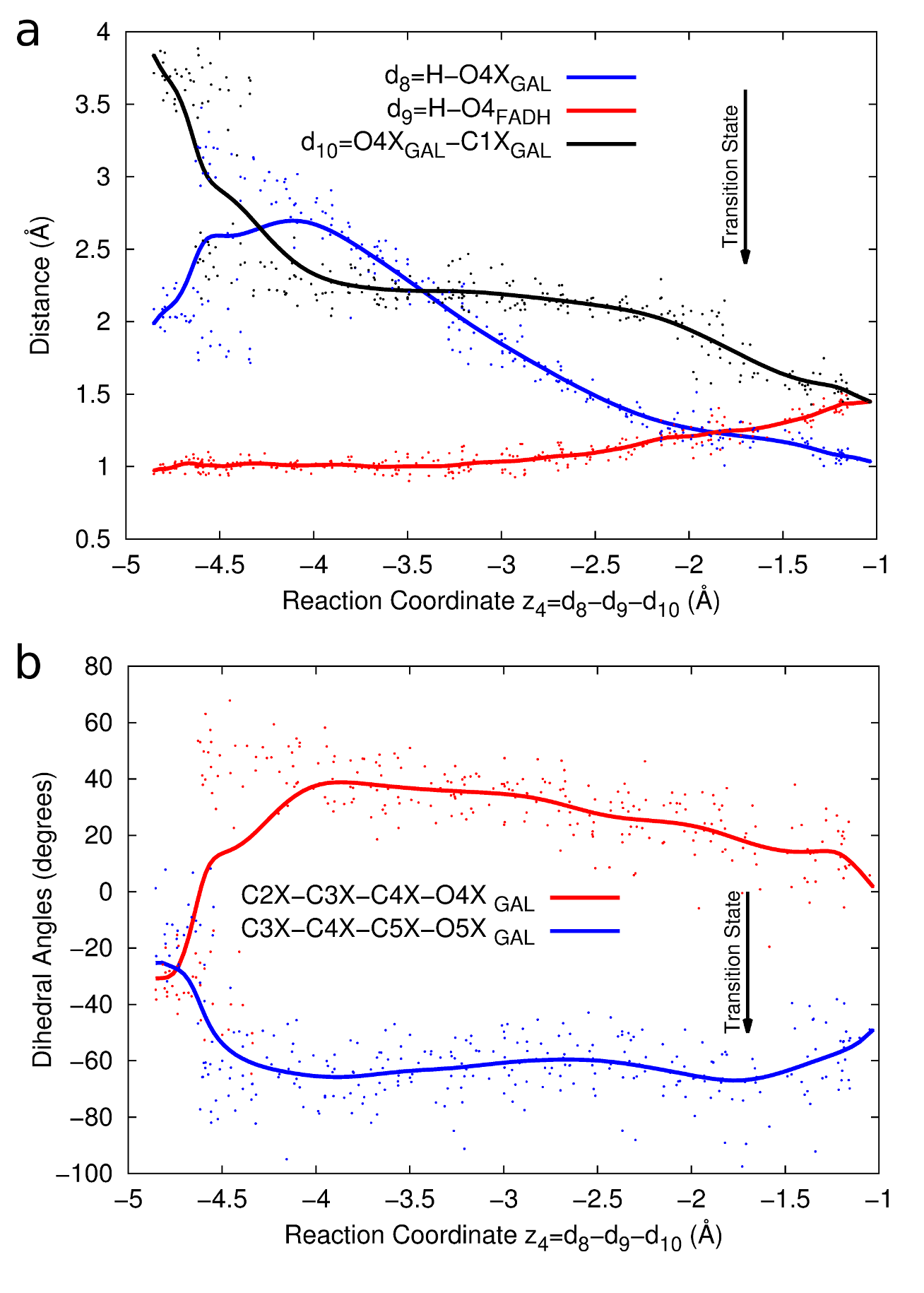

Supplement: Figure S5 — Evolution of important distances and angles along step 4. Panel (a): evolution of the distances involved in the definition of the reaction coordinate . Panel (b): torsional angles that define the orientation of the hydroxyl groups at positions 4 and 5 of galactose. The location of the transition state is indicated with an arrow. (TIFF) [file pone.0109559.s005.tiff]

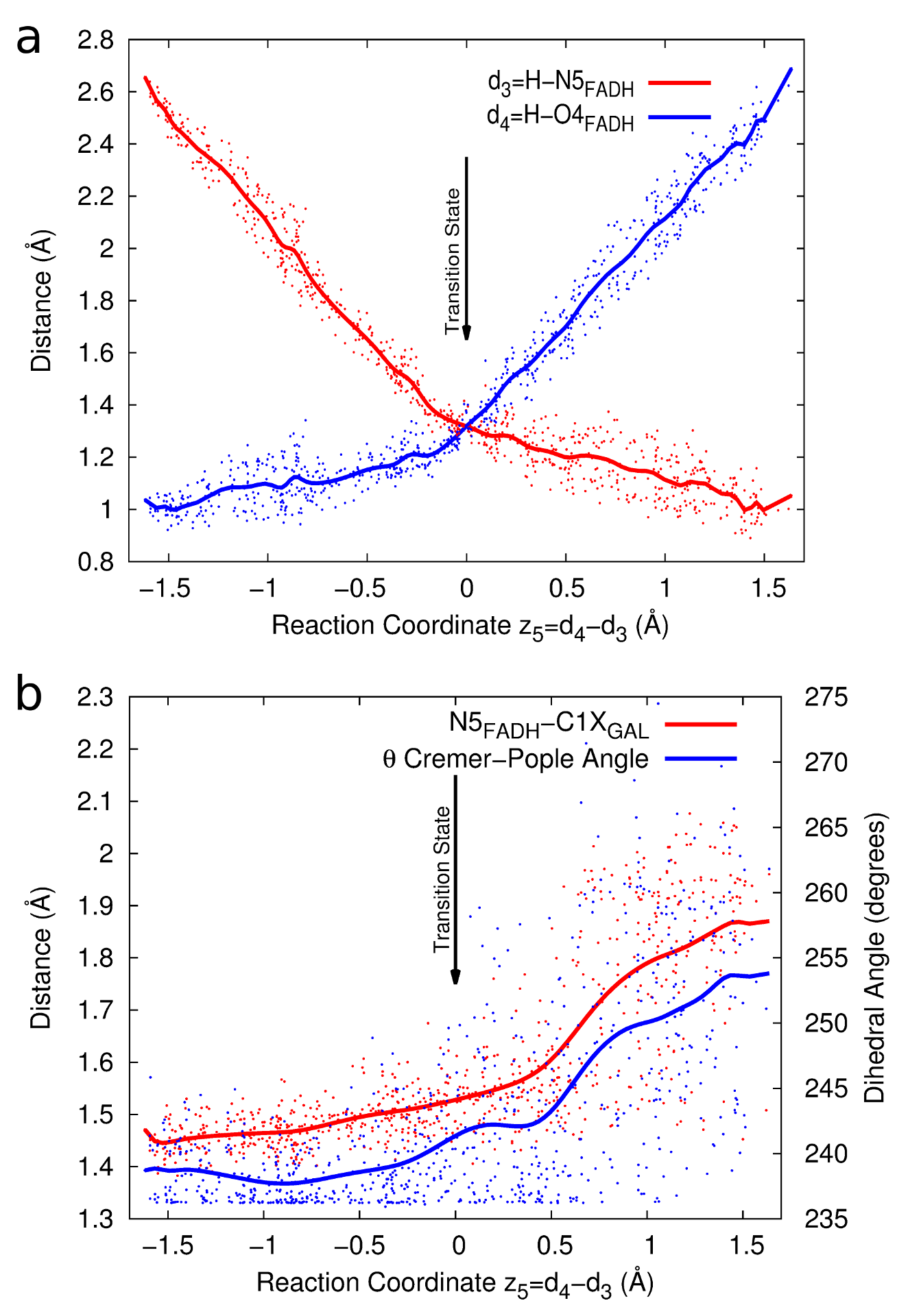

Supplement: Figure S6 — Evolution of important distances and angles along step 5. Panel (a) evolution of the distances involved in the definition of the reaction coordinate . Panel (b) distance between N5FADH and C1XGAL along with the Cremer-Pople angle of furanose. The location of the transition state is indicated with an arrow. (TIFF) [file pone.0109559.s006.tiff]

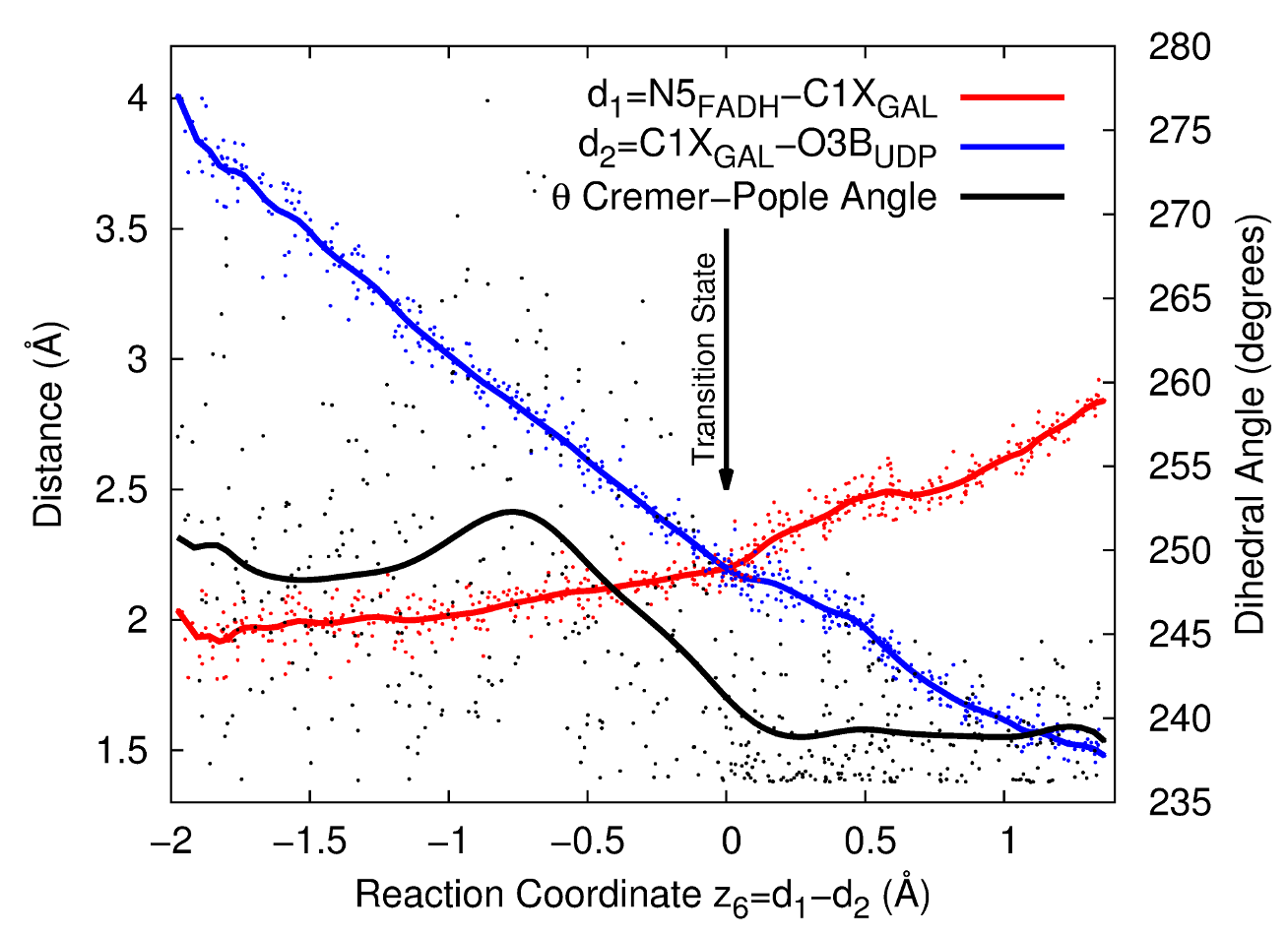

Supplement: Figure S7 — Evolution of important distances and angles along step 6. Evolution of the distances involved in the definition of the reaction coordinate along with the Cremer-Pople angle of the furanose ring. The location of the transition state is indicated with an arrow. (TIFF) [file pone.0109559.s007.tiff]
